# Current Perspectives in the Discovery of Newer Medications Against the Outbreak of COVID-19

**DOI:** 10.3389/fmolb.2021.648232

**Published:** 2021-07-12

**Authors:** M. Ramesh, Krishnan Anand, Mohd Shahbaaz, Magda H. Abdellattif

**Affiliations:** ^1^ Department of Pharmaceutical Analysis, Omega College of Pharmacy, Hyderabad, India; ^2^ Department of Chemical Pathology, School of Pathology, Faculty of Health Sciences and National Health Laboratory Service, University of the Free State, Bloemfontein, South Africa; ^3^ South African Medical Research Council Bioinformatics Unit, South African National Bioinformatics Institute, University of the Western Cape, Cape Town, South Africa; ^4^ Department of Chemistry, College of Science, Deanship of Scientific Research, Taif University, Taif, Saudi Arabia

**Keywords:** coronavirus, ACE-2, spike protein, COVID-19, SARS-CoV-2

## Abstract

A rapid and increasing spread of COVID-19 pandemic disease has been perceived worldwide in 2020. The current COVID-19 disease outbreak is due to the spread of SARS-CoV-2. SARS-CoV-2 is a new strain of coronavirus that has spike protein on the envelope. The spike protein of the virus binds with the ACE-2 receptor of the human lungs surface for entering into the host. Therefore, the blocking of viral entry into the host by targeting the spike protein has been suggested to be a valid strategy to treat COVID-19. The patients of COVID-19 were found to be asymptomatic, cold, mild to severe respiratory illness, and leading to death. The severe illness has been noted mainly in old age people, cardiovascular disease patients, and respiratory disease patients. However, the long-term health effects due to COVID-19 are not yet known. Recently, the vaccines were authorized to protect from COVID-19. However, the researchers have put an effort to discover suitable targets and newer medications in the form of small molecules or peptides, based on *in-silico* methods and synthetic approaches. This manuscript describes the current perspectives of the causative agent, diagnostic procedure, therapeutic targets, treatment, clinical trials, and development of potential clinical candidates of COVID-19. The study will be useful to identify the potential newer medications for the treatment of COVID-19.

## Introduction

### Severe Acute Respiratory Syndrome Coronavirus-2

The virus is an intracellular parasite that affects the susceptible and permissive cells to complete its life cycle. Coronavirus belongs to a large family of zoonotic viruses and mainly affects mammals and birds (Bande et al., 2015; Lee, 2015). A large trimeric crown-like complex of the virus in microscopic observation has given the name, i.e., corona. The genomic structure of coronavirus categorizes them into four subgroups (alpha, beta, gamma, and delta). The alpha and beta subgroups of coronavirus infect humans (Rabi et al., 2020). Coronavirus is the beta subgroup in the family of Coronaviridae (Zheng, 2020). In humans, coronavirus causes a respiratory illness from mild cold to severe respiratory diseases. Six strains of coronavirus were known from the past decade. Recently, a novel coronavirus now named severe acute respiratory syndrome coronavirus-2 (SARS-CoV-2) emerged in December-2019. SARS-CoV-2 is an enveloped, positive-sense single-stranded RNA virus with 30,000 base lengths. It was likely to be originated from the bat and is genetically related to other coronaviruses. The new strain of coronavirus (SARS-CoV-2) differs from the previous strains of coronavirus with the critical amino acid residues at the receptor-binding domain (RBD). These residues help the virus to interact with the host organism.

### Coronavirus Disease-2019

The respiratory illness associated with SARS-CoV-2 was named coronavirus disease-2019 (COVID-19). Then, the new strain of severe acute respiratory syndrome coronavirus (SARS-nCoV) was designated as a causative agent for COVID-19 by World Health Organization (Chen et al., 2020a; Imai et al., 2020). COVID-19 spread among the population rapidly in 2020 and has threatened public health extremely. The outbreak of COVID-19 has begun in Wuhan city, China and it has infected around 163,312,429 people worldwide (Imai et al., 2020). Till now, around 3,386,825 deaths were documented globally [Updated on 18th May-2021]. United States of America, Brazil, China, Italy, Iran, India are the most affected countries (Centers for Disease Control and Prevention, 2020; Grasselli et al., 2020; Kim et al., 2020; Zhu et al., 2020a). Due to the increase in the spread of disease, the public health emergency was announced by the World Health Organization. COVID-19 has been viewed as the worst health crisis of the current century.

### Symptoms

COVID-19 is one of the types of respiratory diseases and it is caused by SARS-CoV-2. The pathogenicity of SARS-CoV-2 is similar to that of other severe acute respiratory syndrome coronavirus (SARS-CoV) (Kuba et al., 2005; Rabi et al., 2020). However, SARS-CoV-2 is structurally different in surface proteins and viral load kinetics (Cevik et al., 2020). Moreover, the specific symptoms related to COVID-19 are not known. The nasal discharge and saliva from the infected person spread this virus infection from human to human. Respiratory droplets, direct contact with the infected patients also transmit this virus (Harrison et al., 2020). SARS-CoV-2 is known to cause illness of varying severity, ranging from mild to severe, and even death by affecting the respiratory organs. The symptom may be observed within 2–14 days after exposure to the virus (Li et al., 2020a). The average incubation period for the development of the disease is 5 days. The initial phase of COVID-19 shows flu-like symptoms, and it is progressed into organ dysfunction at the later phase (Harrison et al., 2020). Fever, dry cough, cough, sore throat, shortness of breath, malaise, respiratory distress, diarrhea, and taste disturbances are the common symptoms of COVID-19 (Jin et al., 2020a). Lymphopenia, thrombocytopenia, dyspnea, septic shock, etc., are symptoms observed at the severe stage of COVID-19 (Centers for Disease Control and Prevention, 2020; Harrison et al., 2020). The respiratory illness, neurological problems, vascular to renal deficiency, the autoimmune response was also observed at the various stages of infection. The complication increases mostly among the older people, the patients associated with cardiovascular disease, diabetes, chronic respiratory problems, cancer, etc., (Grasselli et al., 2020; Wang et al., 2020a).

### Diagnostic Procedure for COVID-19

The increased level of C-reactive protein, lactate dehydrogenase, or both can be observed after 3–6 days of exposure to the virus (Poggiali et al., 2020). The diagnosis of COVID-19 is based on real-time PCR (Jawerth, 2020), Sanger sequencing (Lee et al., 2013; Yu et al., 2020a), NGS genome analysis (Chiara et al., 2020), nucleic acid amplification (Mustafa Hellou et al., 2021), microarray-based assay techniques (Hua et al., 2015) etc., Corman *et al* developed a diagnostic workflow for the detection of a novel coronavirus, i.e., SARS-CoV-2 (Corman et al., 2020). The other diagnostic methods like colorimetric assay based on gold nanoparticles and COVID-19 IgG rapid test kit are also employed for the diagnosis of SARS-CoV-2 (Amawi et al., 2020; Corman et al., 2020; Okamaoto et al., 2020).

The diagnostic methods for the detection of SARS-CoV-2 may be classed into five groups, 1) RT-PCR: RT-PCR is an experimental-based method for the detection of genomic RNA. The process of RT-PCR uses an enzyme reverse transcriptase for the conversion of RNA into complementary DNA, followed by the amplification of cDNA. The procedure uses the respiratory samples for diagnosis. It involves reaction primers and genetically engineered probes for detection (Corman et al., 2020). 2) LAMP/RT-LAMP: The loop-mediated amplification (LAMP) technique involves the amplification of nucleic acid at a single temperature (60–65°C). It is an alternative diagnostic method to PCR for the detection of SARS-CoV-2. The diagnosis can be employed on crude samples with a high amplification rate (10^9^ copies of the gene within an hour) (Yüce et al., 2021). 3) CRISPR technique: CRISPR amplifies the nucleic acid of RNA sequence *via cas variant* (Cas13 for SHERLOCK, Cas12a for DETECTR). The method is rapid and specific (Mohamadian et al., 2021). 4) Chest-CT scan: A chest CT scan requires a specialized instrument to conduct the test. Ground-glass opacities, vascular enhancement fibrosis, and interlobular septal thickening were identified as characteristic features in the diagnosis (Hani et al., 2020). 5) Serology testing (antibody detection): An immune system produces antibodies in response to the antigen. IgA, IgD, IgE, IgG, and IgM are the antibodies and IgM is the antibody produced at the time of infection, IgG is abundant in the blood. These antibodies neutralize antigens by binding to them. The antibody detection tests estimate the concentration of IgM and IgG levels in the blood sample to diagnose the patients (Jacofsky et al., 2020).

## The Treatment for COVID-19 Infection

An ideal strategy to stop the spread of COVID-19 involves the blocking of the cause of infection rather than treating the disease symptoms. The therapeutic strategies for the treatment of SARS-CoV-2 may be classified into the following categories 1) inhibition of viral binding to the host 2) inhibition of viral replication 3) restoring the host’s immunity. The identification of specific drugs based on the above-mentioned classifications would help in the management of COVID-19 infection. However, no selective drugs have been discovered/approved by the Food and Drug Administration (FDA) for the treatment of COVID-19. At present, few vaccines were approved and many others are under clinical trials to treat COVID-19 (Borah et al., 2021). An attempt to repurpose the existing and clinically approved drugs is also in progress (Amawi et al., 2020).

### Vaccination

Sputnik V is the first vaccine against COVID-19 and it was approved in Aug-2020. The vaccine was developed based on two human adenovirus vectors. Adenovirus is a common cold virus and the encoding gene of the spike protein of SARS-CoV-2 generates immunity. The adenovirus type 26 and type 5 are used as vectors to develop the vaccine. Adenovirus type 26 based vaccine is administered on the first day of vaccination and type 5 is administered to boost the immunity on the 21st day. At the time of approval of Sputnik V in Russia, it was declared safe. However, it was also commented as a premature vaccine since the phase-3 trial was not completed. Nevertheless, phase-1 and phase-2 studies have reported 91.6% efficacy of the vaccine and the study has not shown any unusual side effects. Then, Sputnik V has been launched and reached 59 other countries on Apr-2021 (Logunov et al., 2020).

### Convalescent Plasma Therapy

Plasma is the liquid component of blood and it is yellowish in appearance. The plasma separated from blood contains minerals, proteins, and antibodies. The COVID-19 patients recovered from the infection may have developed antibodies to combat COVID-19. Therefore, this approach transfuses these antibodies into COVID-19 patients. The transfused antibody neutralizes foreign objects like SARS-CoV-2. Convalescent plasma therapy is employed as a treatment procedure for critically ill patients of COVID-19. It provides passive immunity to the COVID-19 patients to recover. Convalescent plasma therapy is effective in the absence of antiviral agents or vaccines. It has been approved by FDA in the United States for the management of COVID-19 (Rojas et al., 2020).

### The Chinese System of Medicine

Chinese traditional medicine provides ShuFengJieDu capsules and Lianhua Qingwen capsules for the treatment of COVID-19. The clinical trial data to ensure the safety and efficacy of these Chinese traditional medicines against COVID-19 is not available. However, the therapeutic significance of ShuFengJieDu capsules against respiratory tract infection, and pulmonary infection is known (Chen et al., 2020b; Ji et al., 2020). ShuFengJieDu capsule consists of polygonum cuspidatum, radix isatidis, forsythia, verbena, bupleurum, radix, and reed root. It has shown broad-spectrum antiviral activity against several viruses including influenza-A virus H1N1 and adenovirus (Wang et al., 2020b). Lianhua Qingwen capsule has been used for the treatment of viral influenza, and SARS. The various therapeutic actions of Lianhua Qingwen are antiviral activity, anti-inflammatory activity, and immune system-related functions (Li et al., 2020b). Quercetin, luteolin oxalin, and kaempferol are active ingredients of Lianhua Qingwen. The broad-spectrum activity of Lianhua Qingwen is through the mitogen-activated protein kinase and Hepatitis B signaling pathways (Wang et al., 2020b).

### Existing Drugs

**FIGURE 1:**
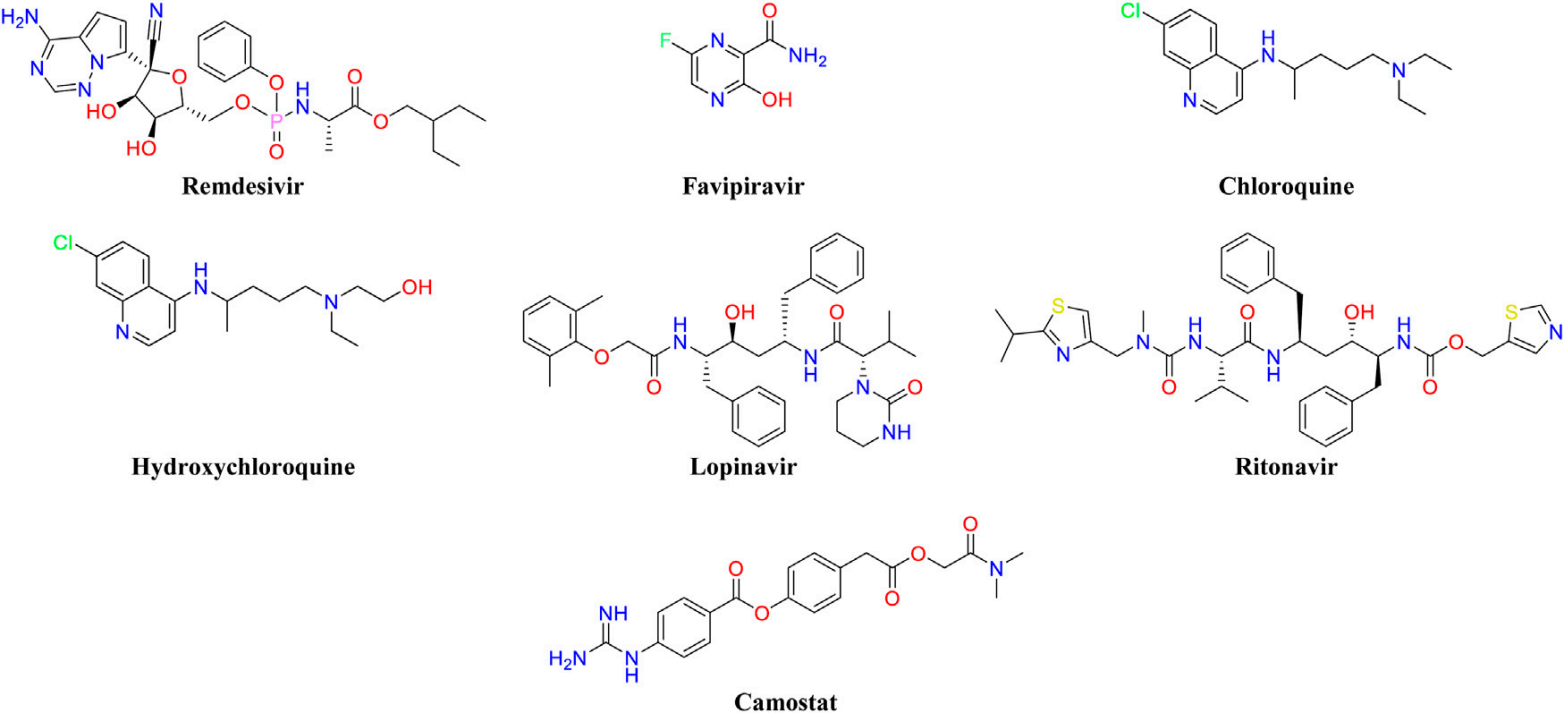
The reported drug molecules showing promising results against COVID-19.

The antiviral agents (remdesivir, favipiravir) (Martinez, 2020; Wang et al., 2020c), antimalarials (chloroquine, hydroxychloroquine) (Xu et al., 2020), and the combination of (lopinavir/ritonavir) (Lu, 2020; Zhang et al., 2020a) have shown promising results and are also in the various stages of attempt for the treatment of COVID-19 (Figure 1) (Amawi et al., 2020; Busse et al., 2020). Remdesivir has shown inhibitor activity against SARS-CoV as well as MERS-CoV, and it is already under clinical trial for the treatment of Ebolavirus and COVID-19 infection (Kotta et al., 2020). Recently, the SARS-CoV-2 controlling activity of remdesivir in *in-vitro* has been reported (Wang et al., 2020c). The preliminary clinical trial data has shown faster recovery from COVID-19 when the patients with moderate to severe symptoms are treated with remdesivir and other supportive therapy (Malin et al., 2020). Chloroquine reduced the viral load in several studies and hydroxychloroquine has shown short-term efficacy in COVID-19 patients (Zhang et al., 2020a). A clinical trial for the combination of lopinavir-ritonavir has been conducted with hospitalized adults of COVID-19 patients. The combined medication of lopinavir-ritonavir (400:100 mg) has been given twice a day for a period of 14-day. Gastrointestinal adverse effects were observed among the treatment groups. A significant therapeutic improvement was not observed with the lopinavir-ritonavir combination and the trial has been stopped (Cao et al., 2020).

### Monoclonal Antibodies

The monoclonal antibodies provide passive immunity to the inhibition of viral entry into the host by various processes like antibody-dependent cellular cytotoxicity, phagocytosis, and antibody-mediated neutralization. Recently, the monoclonal antibodies 1) the combination of casirivimab (REGN10933)-imdevimab (REGN10987) 2) the combination of bamlanivimab (LY-CoV555)-etesevimab were authorized for the emergency use in the treatment of COVID-19 from mild to severe cases by FDA. The combination of bamlanivimab-etesevimab has reduced the hospitalization of COVID-19 patients. Casirivimab-imdevimab combination has been rationalized to control the mutated SARS-CoV-2. Bamlanivimab targets the spike protein of SARS-CoV-2 to prevent viral entry (Wolf et al., 2021). Tocilizumab is used for the handling of rheumatic diseases. It is a type of recombinant IL-6 human monoclonal antibody. Tocilizumab along with other antiviral agents has shown significant improvement among COVID-19 patients in the retrospective study (Zhang et al., 2020a). However, the safety, efficacy, and validity of all these medications against COVID-19 have not yet been confirmed with the extensive clinical trial experiments.

## Therapeutic Targets

**FIGURE 2:**
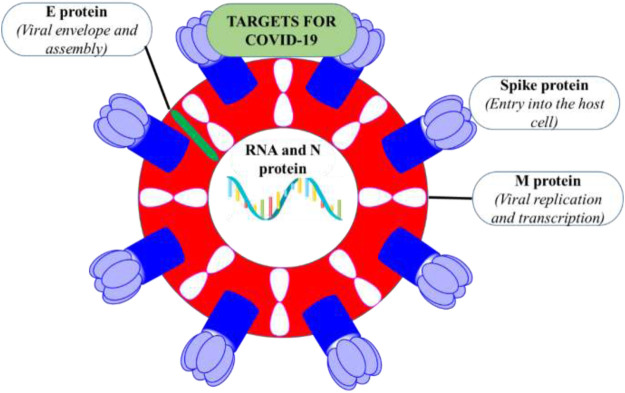
The representation of therapeutic targets for COVID-19 in SARS-CoV-2.

**FIGURE 3:**
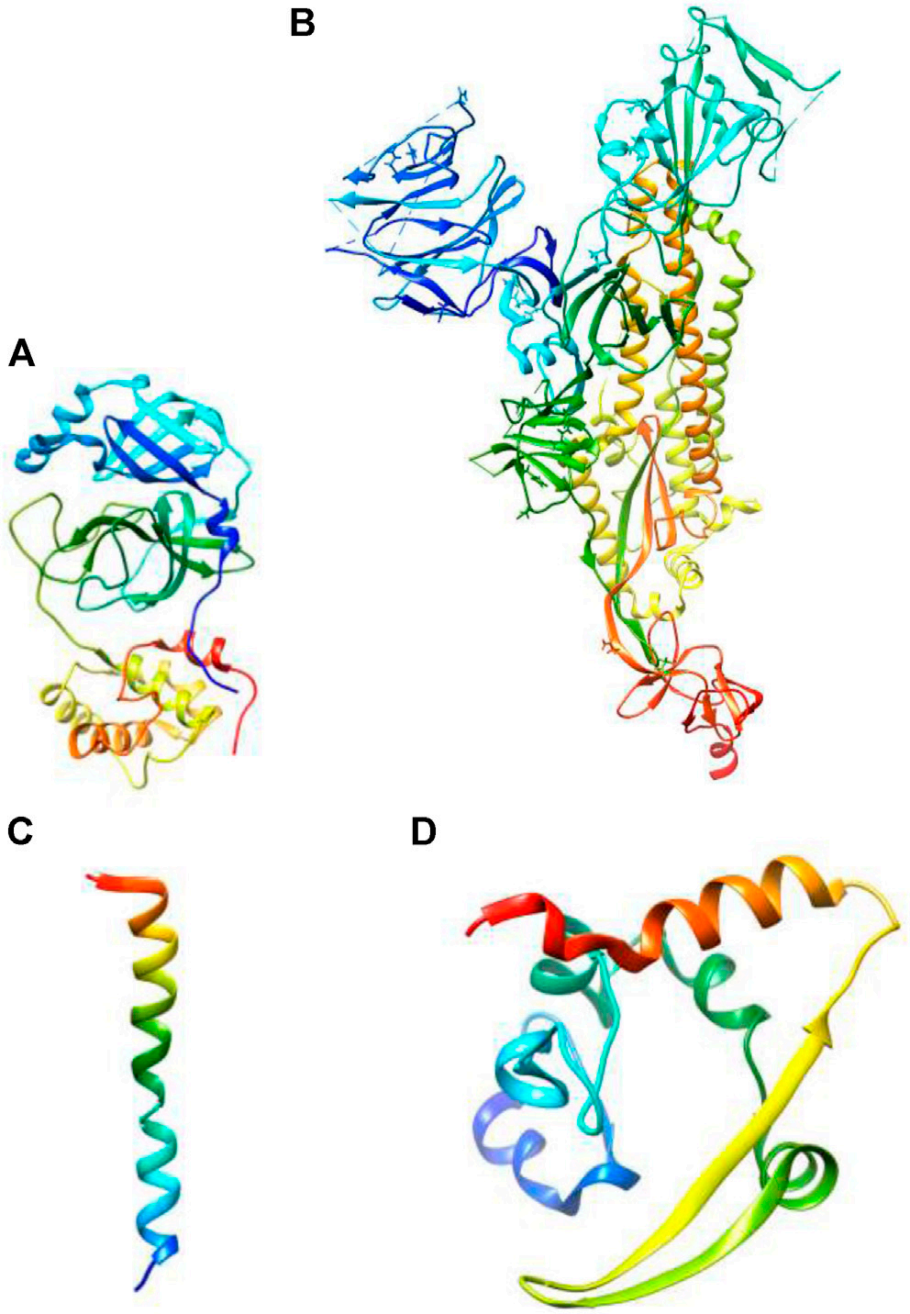
Three-dimensional structures of **(A)** main protease (PDB ID: 7BUY) **(B)** spike protein (PDB ID: 6VXX) **(C)** envelope protein (PDB ID: 7K3G) **(D)** nucleocapsid protein (PDB ID: 6YUN) (The protein is blue at the N-terminus and red at the C-terminus).

The size of SARS-CoV-2 is ∼120 nm in diameter (Lim et al., 2016). The molecular targets for the inhibition of entry of SARS-CoV-2 are 1) M-protein (main protein-M^pro^) 2) S-protein (spike protein) 3) E-protein (envelope protein) 4) N-protein (nucleocapsid protein) (Figure 2). All these proteins are required for the production of complete viral particles (Table 1). The inhibition of these viral proteins affects the virus’s life cycle. Several crystal structures for these therapeutic targets are obtainable from the protein data bank (PDB) (Figure 3). The understanding of the structural features and mechanistic functions of these molecular targets sets the stage to discover a newer medication for the treatment of COVID-19 infection.

**TABLE 1:** The structures and functions of therapeutic targets of COVID-19.

No	Target proteins	Functions	PDB IDs	Resolution	(Ref.)
1	Main protease (M^pro^)	Viral replication and transcription	7BUY	1.60 Å	Jin et al. (2020b)
2	Spike protein (S-protein)	Viral entry into the host cell organ	6VXX	2.80 Å	Walls et al. (2020)
3	Envelope protein (E-protein)	Formation of viral envelope and assembly	7K3G	2.10 Å	Mandala et al. (2020)
4	Nucleocapsid protein (N-protein)	Formation of nucleocapsid	6YUN	1.45 Å	Zinzula et al. (2021)

### Main Protease

Main protease (M^pro)^ is the type of papain-like protease and is involved in the maturation of non-structural protein. The replicating gene of SARS-CoV-2 encodes two polyproteins named PP1a and PP1ab. These polyproteins are processed for proteolytic cleavage by M^pro^ to release functional protein. The functional proteins play a crucial role in viral replication and transcription. Therefore, the inhibition of M^pro^ is an attractive strategy for the treatment of COVID-19. The estimated molecular mass of M^pro^ is ∼33.8 kDa in mass spectrometry. The substrate-binding pocket of SARS-CoV-2 constitutes amino acids Cys145 and His41. The sulfhydryl group of Cys145 reacts with the electrophilic fragment of the substrate leading to form a covalent bond. This covalent modification results in the inhibition of M^pro^. Six compounds (Ebselen, Disulfiram, Tideglusib, Carmofur, Shikonin, and PX-12) were reported to inhibit M^pro^ (Table 1) (Jin et al., 2020b).

### Spike Protein

Spike protein (S-protein) is present on the surface structure of coronavirus that helps for the viral entry into the host cell by binding through angiotensin-converting enzyme 2 (ACE-2) receptor (Jin et al., 2020a). Therefore, ACE-2 could act as an RBD for SARS-CoV-2. The RBD of SARS-CoV-2 spike protein has been targeted to competitively inhibit viral entry. The spike protein (∼180–200 kDa) is present in the glycosylated form. The N-terminus part of the spike protein is positioned at the outside of the viral surface whereas the C-terminus is located at the intramembrane space. The composition of spike protein includes S1-subunit and S2-subunit with a cleavage site for protease between S1 and S2. The S1-subunit of spike protein makes initial contact with the host cell ACE-2 receptor for the initial viral entry. The receptor specificity is achieved from the S1-subunit that forms the RBD. The S2-subunit of the SARS-CoV-2 comprises fusion protein, heptad regions (HR1 and HR2), transmembrane, and a cytoplasmic domain. The S2-subunit is responsible for membrane fusion (Table 1) (Jin et al., 2020a; Walls et al., 2020)

### Envelope Protein

Envelope protein (E-protein) is a small-membrane protein that is responsible for the pathogenesis, formation of the viral envelope, viral assembly, viral budding, and interaction with the host cell. The E-protein forms a homopentameric ion channel known as viroporin. The hydrophobic nature of the transmembrane domain of E-protein oligomerizes to form an ion channel. This ion channel helps the virus to interact with other viral proteins as well as host cell proteins. The absence of E-protein coronavirus has shown reduced viral infection. Therefore, the lack of E-protein virus aids vaccine development. The envelope protein is highly expressed in the infected cells during the replication process and promotes the formation of the viral envelope. The molecular mass of E-protein is ∼8.4–12 kDa comprising 76–109 amino acid residues. The structure of E-protein constitutes hydrophilic terminus (7–12 amino acids), carboxy terminus, and hydrophobic transmembrane domain (25 amino acids) (Table 1) (Mandala et al., 2020).

### Nucleocapsid Protein

Nucleocapsid protein (N-protein) encapsulates the RNA genome of the virus. It is involved in the formation of the nucleocapsid. N-protein also participates in viral replication and viral infection (Table 1). The co-expression of N-protein enhances the production of the viruses further. The main function of N-protein is oligomerization and packaging of the single-stranded viral RNA genome. Moreover, it is highly immunogenic and therefore, it is an attractive target protein for the treatment of COVID-19. The molecular mass of N-protein is in the range of 37.7–51.5 kDs. It is organized as an N-terminal domain, intrinsically disordered domain, and C-terminal domain. The N- terminal domain and-C-terminal domain are responsible for genome packing whereas the intrinsically disordered domain is responsible for the RNA binding activity (Zinzula et al., 2021).

## Research Progress and Strategies for the Development of Newer Medications Against COVID-19

The immune response against the vaccine remains unclear. The administration of neutralizing antibodies has been shown to halt the disease progression. However, the possibilities of re-infection cannot be ignored. At present, few vaccines are approved; and few more vaccines, inhibitors, antibodies, immunity enhancers, etc., are at various stages of development. However, major efforts have been put to develop peptide-based drug candidates to treat COVID-19 infection (Vanpatten et al., 2020). Moreover, the computational studies to explore the mechanistic behavior of the therapeutic targets of SARS-CoV-2 are also under progress.

### Vaccines

In many cases, mRNA-based vaccines have been proposed for infectious diseases and cancers. Recently, a thermostable mRNA vaccine candidate known as ARCoV has been developed by Zhang *et al* for the management of the COVID-19 pandemic. The vaccine is a lipid nanoparticle encapsulated type that encodes the RBD of SARS-CoV-2. The intramuscular vaccination of ARCoV produced neutralizing antibodies in mice and other models. Two doses of vaccination have shown complete protection and the vaccine was found to be stable for a week at room temperature. ARCoV vaccine is in the phase-1 stage of clinical trial evaluation (Table 2) (Zhang et al., 2020b).

**TABLE 2:** Research progress on the therapeutic targets of SARS-CoV-2 to treat the COVID-19 infection.

No	Title of the work	Targets	Outcome	Biological screening	Ref
Vaccines					
1	A thermostable mRNA vaccine against COVID-19	RBD of SARS-CoV-2	ARCoV vaccine candidate	It has shown protection in animal models	Zhang et al. (2020b)
2	SARS-CoV-2 mRNA vaccine development enabled by prototype pathogen preparedness	Spike protein	mRNA-1273 vaccine	It has reduced the viral load 100 fold at the concentration of 0.1 µg	Corbett et al. (2020)
3	Design of a multiepitope-based peptide vaccine against the E Protein of human COVID-19: An immunoinformatics approach	E-protein	YVYSRVKNL, SLVKPSFYV, and LAILTALRL	---	Abdelmageed et al. (2020)
**Inhibitors**					
4	Peptide antidotes to SARS-CoV-2 (COVID-19)	Spike protein	SARS-BLOCK™ - a synthetic peptide scaffolds	Single-micromolar concentration	Watson et al. (2020)
5	Computational design of ACE2-based peptide inhibitors of SARS-CoV-2	RBD	Inhibitors-2, inhibitor-3, and inhibitor-4	---	Han and Král, (2020)
6	Design of potent membrane fusion inhibitors against SARS-CoV-2, an emerging coronavirus with high fusogenic activity	Spike protein	Lipopeptide (IPB02)	Dual split-protein based fusion cell-cell assay (0.025 μM)	Zhu et al. (2020b)
7	Peptide-like and small-molecule inhibitors against COVID-19	M^pro^	Cobicistat, ritonavir, lopinavir, and darunavir	---	Pant et al. (2020)
**Antibodies**					
8	A human monoclonal antibody blocking SARS-CoV-2 infection	Spike protein	47D11 antibody	IC_50_ value: 0.57 μg/ml	Wang et al. (2020d)
9	Potent binding of 2019 novel coronavirus spike protein by a SARS coronavirus specific human monoclonal antibody	Spike protein	Monoclonal antibody (CR3022)	KD value: 6.3 nM	Tian et al. (2020)
**Immunity enhancers or modulators**					
10	The potential of antimicrobial peptides as an antiviral therapy against COVID-19	---	Lactoferrin	---	Elnagdy and Alkhazindar, (2020)
11	Type 1 interferons as a potential treatment against COVID-19	---	Type 1 interferons	---	Sallard et al. (2020)
**Miscellaneous**					
12	SARS-CoV and SARS-CoV-2 main protease residue interaction networks change when bound to inhibitor N3	M^pro^	Identified the conformational changes in one cluster and four residues (131, 175, 182, and 185)	---	Griffin (2020)
13	*In silico* discovery of candidate drugs against covid-19	---	Identified 36-drugs candidates as effective agents against COVID-19	---	Cava et al. (2020)
14	Structural basis of SARS-CoV-2 3CL ^pro^ and anti-COVID-19 drug discovery from medicinal plants	Chymotrypsin-like cysteine protease (3CL^pro^)	Identified 9-hit molecules for the management of COVID-19	---	Tahir ul Qamar et al. (2020)
15	Computational screening of antagonists against the SARS-CoV-2 (COVID-19) coronavirus by molecular docking	Main protease	Luteolin has been suggested as a hit molecule for the specific binding with SARS-CoV-2 main protease	---	Yu et al. (2020b)

The mRNA-1273 vaccine has been expressed from the spike trimer of SARS-CoV-2. The mRNA-1273 vaccine induces the neutralizing antibody and triggers CD8 T cell responses against SARS-CoV-2 in mice. The 0.1 µg concentration of mRNA-1273 reduced the viral load by ∼100 fold and 1 µg of mRNA-1273 was observed to be effective for 3 months to protect from viral replication. Currently, the mRNA-1273 vaccine is in the phase-2 stage of clinical trial and is focused on the phase-3 clinical evaluation (Table 2) (Corbett et al., 2020).

Multiepitope-based peptide vaccine was designed from an immunoinformatics approach and comparative genomic approach for the management of COVID-19 by Abdelmageed *et al.* The envelope protein of novel coronavirus was used as the immunogenic target to design the T-cell epitope-based peptide vaccine. The gene bank files were obtained from NCBI. The binding affinity of the peptides with major histocompatibility complex classes (MHC-I and MHC-II) was estimated from molecular docking in AutoDock. The study concluded YVYSRVKNL, SLVKPSFYV, and LAILTALRL as potential peptides for the vaccine design against COVID-19 (Table 2) (Abdelmageed et al., 2020).

Promising results from the clinical trials of the BNT162b2 (Pfizer/BioNTech) and ChAdOx1 nCoV-19 (Oxford/AstraZeneca) vaccines were obtained. BNT162b2 (Pfizer/BioNTech) is a nucleoside-modified mRNA vaccine. It encodes the spike protein of SARS-CoV-2. The clinical trial of BNT162b2 has begun in April-2020 and it has been tested on 40,000 people. The phase-1 and phase-2 trials reported the safety and efficacy of this vaccine. The phase-3 clinical trial has reported the safety, efficacy, tolerability, and immunogenicity at the mild dose level among the different age groups. Moreover, the vaccination of BNT162b2 (tozinameran) has shown 52.4% efficacy after the administration of the first dose and before the administration of the second dose. After the second dose of vaccination, 94.8% efficacy was observed against COVID-19. Therefore, it has been authorized by the regulatory authority for the emergency use (Oliver et al., 2020).

Several viral-vector-based vaccines are under the various stages of clinical trials (Bezbaruah et al., 2021). ChAdOx1 nCoV-19 vaccine has been developed by Oxford University and AstraZeneca against COVID-19. ChAdOx1 nCoV-19 (AZD1222) is a chimpanzee adenovirus vectored vaccine composed of the coding sequence of the spike protein. The results of phase-3 clinical trials were obtained in November-2020. The overall efficacy of this vaccine is 70.4%. It has been approved by various medicine agencies like the European medicines Agency, Australian Therapeutic Goods Administration, etc., Moreover, the vaccine has closer efficacy as with other vaccines in the low-dose regimen. ChAdOx1 nCoV-19 vaccine may also provide maximum protection against COVID-19 (Knoll and Wonodi, 2021).

### Inhibitors

Watson *et al* demonstrated the role of peptide antidotes (SARS-BLOCK™ - a synthetic peptide scaffolds) to SARS-CoV-2 mediated COVID-19 infection. SARS-BLOCK™ inhibits the RBD of the S1-part of spike protein that binds to ACE-2, the S1-subunit is essential for the SARS-CoV-2 to enter into the host cell through ACE-2. These peptide scaffolds were designed by mimicking the RBD of spike protein using computational technologies like SWISS-MODEL, PDBePISA, and RaptorX. Biomimetic technology has also been applied to enhance the stability of these designed peptides. Then, the peptides were synthesized by solid-phase peptide synthesis technology. The synthetic peptides were characterized for their binding to ACE-2 in biolayer interferometry. The peptides have shown single-micromolar affinities to ACE-2. Therefore, it may serve as a novel prophylactics as well as immune stimulants against COVID-19 (Table 2) (Watson et al., 2020).

The peptide inhibitors were designed to block the RBD of SARS-CoV-2 based on ACE-2. The conformation and stability of the peptides were analyzed by molecular dynamics in NAMD software. The peptides which use α_1,2_ helices have shown bent shape in simulation studies. This conformation matches with the RBD domain of SARS-CoV-2. The amino acid residues of ACE-2, i.e., 24(Q), 27(T), 30(D), 31(K), 34(H), 35(E), 37(E), 38(D), 41(Y), and 42(Q) of *α*1; 82(M) of *α*2; 353(K), 354(G), 355(D), and 357(R) of the linkering unit between *β*3 and *β*4 were found as interacting residues with RBD. The study designed four types of inhibitors based on the amino acid residues 1) inhibitor-1 (*α*1-helix), 2) inhibitor-2 (*α*1-and *α*2-helices), 3) inhibitor-3 (*α*1, *α*2, and *β*3, *β*4) and 4) inhibitor-4 (same as inhibitor-3 with a different linker). The approach proposed these inhibitors as efficient therapeutic candidates for COVID-19 (Table 2) (Han and Král, 2020).

The entry of SARS-CoV-2 into the human occurs through the fusogenic action of the spike protein of the virus with the human ACE-2 receptor. Therefore, the membrane fusion inhibitors (IPB01and IPB02) with higher fusogenic activity have been designed against SARS-CoV-2 by Zhu et al. The fusogenic inhibitor IPB02 has been designed using the sequence of HR2 in spike protein. IPB02 is a lipopeptide that has shown significant thermal stability and binding affinity. The synthetic peptide IB02 exhibited cell-fusion inhibition at the level of 0.025 μM in a dual split-protein-based fusion cell-cell assay. The study has described the entry pathway of SARS-CoV-2 into the host and the design of fusion inhibitors (Table 2) (Zhu et al., 2020b).

Peptide-like and small molecules were predicted as potential inhibitors for COVID-19 using *in-silico* methods from the databases of CHEMBL, ZINC, FDA-approved drugs, and effective molecules under clinical trials. The study employed molecular docking, molecular dynamics analysis using the crystal structures (PDB ID: 6Y2F, PDB ID: 6W63) to identify the potential candidates to inhibit M^pro^. The potency of the hit molecules was analyzed based on docking scores and binding affinities. Cobicistat, ritonavir, lopinavir, and darunavir were predicted as potential inhibitors for M^pro^. However, the experimental validation of the computational results was not documented (Table 2) (Pant et al., 2020).

### Antibodies

The first human monoclonal antibody to block SARS-CoV and SARS-CoV-2 infection in cell culture has been reported. The recombinantly expressed 47D11 antibody has shown cross-neutralizing activity against SARS-CoV-2. It binds with the spike protein of SARS-CoV-2 and its binding was characterized by immunofluorescence microscopy. The antibody can also be used for the detection of antigens (Table 2) (Wang et al., 2020d).

A SARS-specific human monoclonal antibody for binding with novel coronavirus spike protein has been reported. The antibody was obtained from the bloodstream of the SARS patient. The monoclonal antibody (CR3022) has shown potent binding with RBD of SARS-CoV-2 (KD value: 6.3 nM). The other SARS-specific neutralizing antibodies (m396, CR3014) did not show a significant binding affinity with the spike protein of novel coronavirus. Therefore, the study indicated the difference between the RBD of SARS-CoV and SARS-nCoV. The study suggested that the monoclonal antibody (CR3022) may be served alone or along with other therapeutics to stop the COVID-19 infection (Table 2) (Tian et al., 2020).

### Immunity Enhancers or Modulators

The antimicrobial peptides possess a wide range of antiviral activities from human to animal viruses. The antimicrobial peptide molecules are small in size consisting of 10–100 amino acids. These are amphiphilic with a cation charge that helps to attach to viruses. Lactoferrin is one of the antimicrobial peptides that has been proposed as an immunity enhancer by Elnagdy *et al.* Since lactoferrin has shown immunity enhancement against viral infection, it has been proposed as a promising candidate for the treatment of COVID-19. Lactoferrin is usually present in breast milk and the mucosal layer (Table 2) (Elnagdy and Alkhazindar, 2020).

Type-1 interferons are mainly secreted by plasmacytoid dendritic cells when it identifies the viral components. Type-1 interferons have also shown a wide-ranging of antiviral activities and it is under clinical trial against the MERS-CoV. INF-1 is the first cytokine produced during the initial pathogenesis of viral infection. It interferes with the viral replication process and promotes adaptive immunity. Based on the structural resemblance of MERS-CoV with SARS-CoV, the possibilities of type-1 interferon for the handling of COVID-19 have also been proposed (Table 2) (Sallard et al., 2020).

### Miscellaneous

The residue interaction networks change in the M^pro^ of SARS-CoV and SARS-CoV-2 due to inhibitor binding has been studied by Griffin *et al.* The network clustering was performed with and without inhibitor (N3) in M^pro^ of SARS-CoV and SARS-CoV-2. The network change among the cluster of residues (17, 18, 30–33, 70, 95, 98, 103, 117, 122, and 177) has been observed when the inhibitor N3 binds with M^pro^. Moreover, the amino acid residues (131, 175, 182, and 185) were found to be responsible for the conformational changes. The study revealed the conformational changes of M^pro^ when binding with the inhibitor and provided the structural insights to develop an inhibitor for M^pro^ (Table 2) (Griffin, 2020).

Cava and co-workers carried out *in-silico* discovery to investigate the molecular mechanism of ACE-2 with COVID-19 and found few candidate drugs against COVID-19. The study reported 36 drugs including nimesulide, fluticasone propionate, thiabendazole, photofrin, didanosine, and flutamide as potential candidates for COVID-19 from the gene expression analysis. However, the experimental validation of the results has not been conveyed (Table 2) (Cava et al., 2020).

The replication process of SARS-CoV-2 in the life cycle is mainly controlled by 3-chymotrypsin-like cysteine protease (3CL^pro^). Therefore, 3CL^pro^ has been recognized as a therapeutic target for COVID-19. Qamar *et al* constructed a 3D structural model for 3CL^pro^ from the sequence analysis and studied the structural basis of 3CL^pro^. Then, the *in-silico* screening was carried out on the library of medicinal plant compounds based on molecular docking, ADME prediction, and molecular dynamics. The approach results in the identification of nine hit molecules that could serve as potential therapeutic candidates for COVID-19 (Table 2) (Tahir ul Qamar et al., 2020).

Ribavirin, remdesivir, chloroquine, and luteolin were computationally screened using AutoDock software to study the binding mechanism toward SARS-CoV-2. Luteolin and chloroquine have shown substantial binding affinity at the binding site of SARS-CoV-2 main protease. Luteolin has shown the binding interaction with Gln189, Leu4, Asn142, Thr26, Met49, and Val3 residues. These interactions were found to be as similar as N3 which is a bound ligand of the main protease. Luteolin has been projected as a possible hit molecule for the specific binding with SARS-CoV-2 main protease (Table 2) (Yu et al., 2020b).

## Conclusion

There is a serious requirement to develop newer drugs, vaccines, and therapy for the treatment of COVID-19. The approaches based on traditional drug discovery are slow, time-consuming, and costly. Therefore, traditional drug discovery approaches may not be an appropriate method to discover newer medications during the outbreak of COVID-19. The computer-aided drug discovery approaches intending to repurpose the existing and approved drugs or the development of peptide-based drugs may provide a rapid solution against COVID-19. Although, the peptide-based drug candidates are preferable to develop in terms of time, cost, specificity, and affinity; the proteolytic instability of peptide-based drugs would be a major concern in the development processes. Therefore, the approaches based on drug-repurposing, and development of peptidomimetic candidates may be effective to discover newer medications during the COVID-19 outbreak. The information provided in this article may shed a light to discover newer medications to eradicate the COVID-19.
